# Clustering of pediatric onset inflammatory bowel disease in Finland: a nationwide register-based study

**DOI:** 10.1186/s12876-022-02579-1

**Published:** 2022-12-12

**Authors:** Atte Nikkilä, Anssi Auvinen, Kaija-Leena Kolho

**Affiliations:** 1 Faculty of Medicine and Health Technology, Tampere University, Tampere, Finland; 2 Faculty of Social Sciences, Tampere University, Tampere, Finland; 3 Children’s Hospital, Pediatric Research Center, University of Helsinki and HUS, Stenbäckinkatu 11, 00029 Helsinki, Finland; 4 Department of Pediatrics, Tampere University Hospital, Tampere, Finland

**Keywords:** Cluster analysis, Crohn’s disease, Environment, Epidemiology, Ulcerative colitis

## Abstract

**Background:**

The incidence of pediatric inflammatory bowel disease (PIBD) has increased dramatically during the past decades. This implies involvement of environmental factors in etiology but lends no clues about specific agents. We evaluated clustering in time and place of residence at PIBD onset using a case-control setting with comprehensive nationwide register data.

**Methods:**

We included all PIBD cases diagnosed at ages < 18 years during 1992–2017 (3748 cases; median age of 14.6; 2316 (58%) with ulcerative colitis (UC), 1432 with Crohn’s, and 18,740 age- and sex-matched controls) and constructed complete residential histories (including coordinates) from the national database until the date of the diagnosis of the case assigned as index date for the controls. Using the coordinates of the addresses of the subjects and the diagnosis/index dates, we evaluated clustering in time and place using the Knox test. Four temporal (2, 4, 6, 12 months) and four distance (0.25, 0.5, 1, 5 km) thresholds were used, and results were calculated separately for Crohn´s disease and UC. Similar analyses were conducted using the addresses at birth and the addresses five years before the diagnosis or index date. Based on the threshold values displaying the most clustering in the Knox test, logistic regression models were built to identify whether sex, age at diagnosis or the year of diagnosis affected the probability of belonging to a cluster. To analyze clustering in time and place throughout the residential histories, we used Jacquez’s Q with an open-access python program *pyjacqQ*.

**Results:**

The mean number of residencies until the index date was 2.91 for cases and 3.05 for controls (*p* = 0.0003). Knox test indicated residential clustering for UC with thresholds of 500 m between locations and time-period of four months (*p* = 0.004). In the regression analysis, sex, age at diagnosis or year of UC diagnosis did not show differences between the clustered and other cases. Jacquez Q analyses showed higher than expected frequency of clustered cases throughout residential histories (*p* < 10^− 8^).

**Conclusion:**

Our findings suggest that the incidence of PIBD, especially of UC, exhibits clustering in locations of residencies over time. For the clustered cases, environmental triggers warrant future studies.

**Supplementary Information:**

The online version contains supplementary material available at 10.1186/s12876-022-02579-1.

## Background

During the past decades, the incidence of pediatric inflammatory bowel disease (IBD), including Crohn’s disease (CD), ulcerative colitis (UC) and unclassified colitis (IBDU), has dramatically increased in several Western countries [1–3]. IBD pathogenesis involves genetic predisposition in conjunction with dysregulated immune responses and alterations in the gut microbiome [4]. Environmental factors are bound to play a role in such a rapid increase in incidence, but so far, no specific exposures have been clearly identified. The annual incidence of pediatric onset IBD (PIBD) reached 23/100,000 in Finland in 2011–2014 [2]. This is higher than the figures reported from North America (15.2/100,000) and in Asia/the Middle East and Oceania (11.4/100,000) [1].

Although most studies evaluating incidence of PIBD report increasing trends, some have shown stable rates [1]. Geographic variation with higher incidence (or prevalence) in the north than in the south has been reported e.g. in Finland and in the US [5, 6]. Also, migration to a high-incidence country may carry an increased risk [7, 8]. Previously, we reported higher incidence rates of PIBD in the districts with low compared to high density of child population but were unable to identify environmental exposures [5].

In this study, we analyzed clustering of PIBD in time and place and its subtypes in Finland based on nationwide registry data with a case-control setting to improve the understanding of factors underlying the changes in PIBD incidence.

## Subjects and methods

In this register-based case-control study, we identified all cases with PIBD diagnosed at < 18 years of age in Finland during 1992–2017 from the drug reimbursement registry of the Social Insurance Institution (SII). Finland has a comprehensive national health insurance scheme that covers reimbursements for the costs for prescribed medications. Eligibility for higher reimbursement in defined diseases including IBD requires a medical certificate verifying that the diagnosis is appropriately confirmed, primarily by a gastroenterologist, a pediatrician or a surgeon. The ICD-10 diagnosis codes K50 and K51 were included in the registry to separately identify patients with CD and patients with UC (with the latter code including IBDU). A trained professional at the SII evaluates the compliance with the criteria. The benefit is granted retrospectively from the date the certificate was issued. Thus, we used this date as a proxy for the date of diagnosis and as the index date for the matched controls. The excellent coverage and validity of the registry for PIBD was previously demonstrated [9, 10].

We identified five age- and sex-matched controls for each patient from the Digital and Population Data Services Agency (DVV). The matching criterion for age was +/-6 months. Complete residential histories with accurate coordinates were constructed for all study subjects from DVV. The Finnish Population Information System contains basic information on all permanent residents of Finland, linked by a unique personal identity code.

### Statistical analysis

The annual differences in the means of the geographical latitudes for the place of residence between cases and controls were plotted and smoothed curves were fitted for illustration. To analyze clustering solely in place, we identified the five nearest neighbors of each case at index date and compared the observed to the expected ratio of cases and controls among the nearest neighbors. This was repeated for three time periods (1992–2000, 2000–2008, 2008–2017), and separately for CD and UC.

Using the coordinates of the addresses at the time of the diagnosis and the diagnosis dates, we evaluated clustering in time and place using the Knox test (see Supplement for detailed description). Four temporal (2, 4, 6, 12 months) and four distance (0.25, 0.5, 1, 5 km) thresholds were used, and analyses were performed separately for CD and UC. Similar analyses were conducted using the addresses at birth and the addresses five years before the diagnosis date. Subjects who had resided only in a single dwelling were also analyzed separately. Based on the threshold values displaying the most clustering in the Knox test, logistic regression models were built to identify whether other available factors (sex, age at diagnosis or the year of diagnosis) affected the probability of belonging to a cluster in time and place.

To analyze clustering in time and place throughout the residential histories, we used Jacquez’s Q with an open-access python program *pyjacqQ* [11]. Both binomial and false detection rate -based approaches were used to correct for multiple testing. As test parameters, we used 15 neighbors and 9,999 iterations.

Statistical analyses were carried out using R (v. 3.6.2, R core team, 2018, Vienna). The reported p-values are two-tailed and p < 0.05 was considered statistically significant. The Benjamini-Hochberg method was used for multiplicity correction. A more detailed description of the statistical methods is available as an additional file (Additional file 1).

## Results

The case series comprised 3748 PIBD cases, 2316 (58%) with UC and 1432 (36%) with CD and 18,740 age- and sex-matched controls. Accurate diagnostic codes were missing for 213 (5.7%) of the cases and they were diagnosed before 2000. The median age at UC diagnosis was 14.8 years (IQR 11.6 to 16.7) and for CD 14.2 years (IQR 11.5 to 16.4). Most of the patients were boys: 60% (n = 862) in CD and 52% (n = 1214) in UC. The mean number of residencies occupied before the index date was 2.91 for cases and 3.05 for controls (p = 0.0003, Wilcox test). In total, 3.9% of all dwellings in the residential histories lacked coordinates.

The Knox test indicated clustering for UC with the lowest *p*-value (0.004) for 500 m distance between locations and 4-month thresholds of time periods (Table 1). In total, 52 UC cases (2.2%) belonged to such clusters and most were living in suburbs or centers of distinct municipalities. In the regression analysis, sex, age at diagnosis or year of UC diagnosis did not show differences between the clustered and other cases. In CD, the analyses showed some evidence for clustering with 1000 m and 4-month thresholds (p-value 0.021 after correcting for multiple testing).

**Table 1:** Clustering of cases with pediatric inflammatory bowel disease in time and place in Finland

	2 months		4 months		6 months		12 months	
	*UC*	*CD*	*UC*	*CD*	*UC*	*CD*	*UC*	*CD*
250 m	0.007*	0.171	0.015*	0.082	0.044*	0.156	0.159	0.272
500 m	0.007*	0.222	0.004*	0.142	0.004*	0.171	0.007*	0.171
1000 m	0.096	0.171	0.096	0.021*	0.096	0.122	0.112	0.127
5000 m	0.071	0.727	0.096	0.515	0.179	0.764	0.134	0.555

Analyses were performed with Knox test using the coordinates of the addresses at the time of the diagnosis and the time intervals to the diagnosis dates. Corrected p-values from Knox test for pediatric ulcerative colitis (UC) and Crohn’s disease (CD) are shown

The *p*-values were calculated using Poisson approximation and they were corrected for multiple testing using the Benjamini–Hochberg method for ulcerative colitis and Crohn’s disease separately

*Statistically significant *p *values (*p* < 0.05) were underlined and marked with an asterisk

**Fig. 1:**
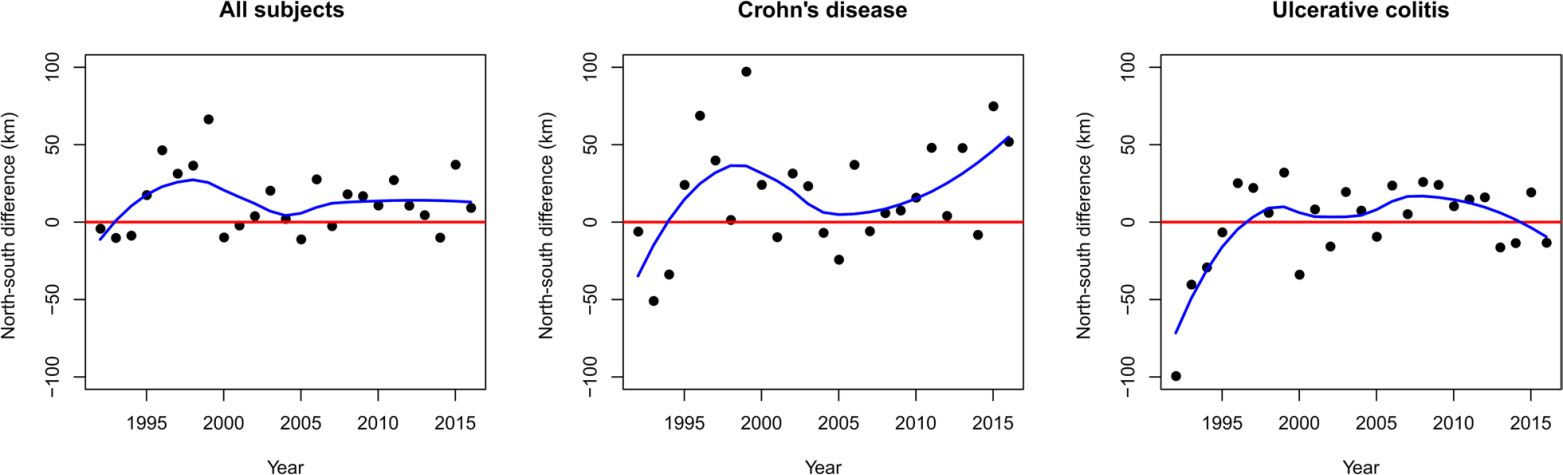
South-North progression of childhood inflammatory bowel diseases in Finland. The first panel considers all subtypes and the second and third consider Crohn’s disease and ulcerative colitis (UC), respectively. All subjects include all patients with UC, Crohn´s diseases and those with no subtype definition. The annual mean values of the geographical latitudes indicating north-south locations of the residencies of cases and controls were compared. A smoothed curve was fitted using local polynomial regression to help with interpretation. Value zero is given when there is no difference in the mean coordinates of the cases compared to controls. Values higher than zero indicate case residencies toward north relative to the control, and values below zero vice versa

Addressing only the spatial component, we found no evidence of clustering in PIBD when considering the five nearest neighbors in either UC or CD overall or during different time periods. We observed no incidence shift toward the north for either of the subtypes (Fig. 1).

In analysis of clustering in time and place, we observed higher than expected proportion of significant findings (*p* < 10^− 8^) for both UC (observed 8.3% vs. expected 5%) and CD (observed 9.4% vs. expected 5%) with Jacquez Q test after binomial correction for multiple testing.

No significant clustering regarding specific time periods (Q_t_) was observed. Using the available hardware, our *p*-value resolution with 9999 iterations was not sufficient to identify significant local clustering (Q_it_), even though the computing time was 21 days for each batch of the final analyses.

## Discussion

We analyzed clustering in time and place of PIBD and its subtypes in a nationwide case-control study based on comprehensive Finnish registries to search for clues of the increasing numbers of patients diagnosed during the past decade or so. Intriguingly, we detected clustering for UC, but not as much for CD (based on the Knox test). The UC clusters were defined by a maximum 500 m difference in residential address and a maximum difference of 4 months in the time of diagnosis. However, the absolute number of clustered cases of UC was low (2% of all UC). Unfortunately, we did not have access to environmental characteristics, e.g. epidemic infections that might have occurred within these residential areas. Also, we did not have access to the medical records of the patients to assess their disease characteristics. In the Jacquez-Q analysis, we found support for these results and observed signs of clustering in time and place for both UC and CD. When studying solely the spatial component, in terms of five nearest neighbors, the analysis did not reveal any indications of clustering. However, this approach is crude in comparison to the above-mentioned Knox test and the Jaquez’s Q method providing results supporting clustering. Also, we observed no signs of shift of new cases towards northern locations during the study period.

To our knowledge, clustering in time and place has not previously been reported for PIBD. A cluster suggests that locally shared etiological factors could affect PIBD occurrence creating an aggregate of cases. The threshold values for UC (4 months, 500 m) could indicate shared environmental exposures, and for example an infection would be a plausible common factor. The main strength of the study is the comprehensive, nationwide register-based data. We identified the patients with PIBD in a register, in which the diagnostic criteria of the patients are confirmed in a medical certificate, further improving the accuracy of the data.

The main shortcoming was the inability to compute Jacquez’s Q statistics for individual clusters (Q_it_) as the current version of the *pyjacqQ* software did not support multi-core computations, which would have allowed us to use high-powered computing servers instead of single high-clock speed cores. It has been suggested that, using Jacquez’s Q method, important clusters could be identified by evaluating clustering events of the subjects with overall clustering, and this approach should be employed in future studies [12].

## Conclusion

The incidence of PIBD, especially of ulcerative colitis, exhibits clustering in locations of residencies over time. Further studies should delve deeper into the disease characteristics and environmental factors of the clustered cases.

## Supplementary information

**Additional file 1.** Supplemental statistical methods.

## Data Availability

The datasets generated during and analyzed during the current study are not publicly available due to general data protection regulation but are available from the corresponding author on reasonable request.

## Abbreviations

CD: Crohn’s disease
IBD: inflammatory bowel disease
IBDU: inflammatory bowel disease unclassified
PIBD: pediatric onset inflammatory bowel disease
UC: ulcerative colitis
