# Supplementary material for: Clustering of pediatric onset inflammatory bowel disease in Finland: a nationwide register-based study
Source: BMC Gastroenterol. 2022 Dec 12;22:512. doi: 10.1186/s12876-022-02579-1 (PMC9743626; doi:10.1186/s12876-022-02579-1)
Supplement: Supplementary file 1 — Additional file 1. Supplemental statistical methods. [file 12876_2022_2579_MOESM1_ESM.pdf]

Supplemental statistical methods

South to North spreading of childhood IBD:
The annual differences in the means of the latitudes (Y-coordinates) of the residencies between cases and controls were compared and plotted. We added smoothed curves to help with the interpretation. In a case of South to North spreading of the childhood IBD, we would observe a slope approaching zero in the figure, as the cases, in the beginning, would have lived more in the Southern parts of Finland, and then would have reached a similar distribution in whole Finland compared to the controls.

Only geographical clustering of childhood IBD:
To analyze solely clustering in place and overlooking the time of the diagnoses, we identified the five nearest neighbors for each case at their diagnosis location. Then we analyzed whether we observed cases and controls in the expected proportion (1:5). If we observe that the proportion of cases is above this, that suggests that cases tend to cluster together even without considering the dates of the diagnoses. We tested this approach for three time periods: 1992–2000, 2000–2008, 2008–2017 and analyzed CD and UC separately.

Knox test for geographical and time clustering of childhood IBD:
Using the coordinates of the diagnosis addresses and the diagnosis dates, we evaluated clustering in time and place, i.e. geographical clustering in locations of residencies over time, using the Knox test. Knox test is designed to look at the interaction of locations and moments in time of the events of interest, in our case, the diagnosis date of childhood IBD. The user of the test needs to specify threshold values for both time and geographical distance. Pairs of cases with a distance below the given threshold are considered close in location and, respectively, the pairs of cases with a time difference below the given threshold are close in time. The test then determines whether being close in location has also an effect on being close in time. If such association is found, this suggests a degree of clustering of space and time. Knox test considers only the cases and thus no comparison to controls is made. (Knox E. & Bartlett M.S.,1964)

Four thresholds for time (2, 4, 6, 12 months) and four for distance (0.25, 0.5, 1, 5 km) were used, and results were calculated separately for CD and UC. Similar analyses were conducted using the addresses at birth and the addresses five years before the diagnosis or index date. Subjects who had resided only in a single dwelling were also analyzed separately.

Logistic regression for the clustered cases identified with Knox test:
logistic regression model was built to identify whether other available factors (sex, age at diagnosis or the year of diagnosis) affected the probability of belonging to a cluster in place and time (i.e. spatio-temporal cluster), defined by the threshold values displaying the most clustering in the Knox test. A similar approach has been used in the context of childhood leukemia with good success. (Kreis C. et al, 2017)

Geographical and time clustering considering all residencies prior diagnoses:
To analyze spatio-temporal clustering throughout the residential histories, we used Jacquez's Q with an open-access python program *pyjacqQ*. (Jirjies S. et al, 2016; reference 10 in the manuscript)

Jacquez's Q method considers the locations of all study subjects (i.e. cases and controls), each time any one of them changes residency. For each of these time slices, the nearest neighbors are determined and then the count of cases among these nearest neighbors is recorded. The method produces multiple statistics: local (Q_{it}), individual (Q_i) and time (Q_t) and their interpretation has been previously clearly described (Table 1 in Sloan et al. 2012 copied below (reference 11 in the manuscript)), We also take note whether the proportion of clustered cases is higher than expected.

With *pyjacqQ*, global, individual and local statistics (according to Jacquez Q) were calculated, and both binomial and false detection rate -based approaches were used to correct for multiple testing. As parameters for the program, we used 15 neighbors and 9,999 iterations.

Statistical analyses were carried out using R (v. 3.6.2, R core team, 2018, Vienna). The reported p-values are two-tailed and $p<0.05$ was considered statistically significant. The Benjamini-Hochberg method was used for multiplicity correction.

Supplemental references:

Jirjies, S., Wallstrom, G., Halden, R. et al. pyJacqQ: Python Implementation of Jacquez's Q-Statistics for Space-Time Clustering of Disease Exposure in Case-Control Studies. J Statistical Software 2016; 74, 1–19. ***reference 10 in the manuscript***

Knox, E. G., & Bartlett, M. S. (1964). The Detection of Space-Time Interactions. Journal of the Royal Statistical Society. Series C (Applied Statistics), 13(1), 25–30.

Kreis C, Lupatsch JE, Niggli F, Egger M, Kuehni CE, Spycher BD; Swiss Paediatric Oncology Group and the Swiss National Cohort Study Group. Space-Time Clustering of Childhood Leukemia: Evidence of an Association with ETV6-RUNX1 (TEL-AML1) Fusion. PLoS One. 2017 Jan 27;12(1):e0170020.

Sloan CD, Jacquez GM, Gallagher CM, Ward MH, Raaschou-Nielsen O, Nordsborg RB, Meliker JR. Performance of cancer cluster Q-statistics for case-control residential histories. Spat Spatiotemporal Epidemiol. 2012 Dec;3(4):297-310. ***reference 11 in the manuscript***
